# *Pseudomonas aeruginosa*, *Staphylococcus aureus*, and Fluoroquinolone Use

**DOI:** 10.3201/eid1108.050116

**Published:** 2005-08

**Authors:** Conan MacDougall, Spencer E. Harpe, J. Patrick Powell, Christopher K. Johnson, Michael B. Edmond, Ron E. Polk

**Affiliations:** * Virginia Commonwealth University, Richmond, Virginia, USA;; † Idaho State University, Boise, Idaho, USA

**Keywords:** fluoroquinolones, Staphylococcus aureus, Pseudomonas aeruginosa, pharmacoepidemiology, drug resistance, microbial

## Abstract

Few long-term multicenter investigations have evaluated the relationships between aggregate antimicrobial drug use in hospitals and bacterial resistance. We measured fluoroquinolone use from 1999 through 2003 in a network of US hospitals. The percentages of fluoroquinolone-resistant *Pseudomonas aeruginosa* and methicillin-resistant *Staphylococcus aureus* (MRSA) were obtained from yearly antibiograms at each hospital. Univariate linear regression showed significant associations between a hospital's volume of fluoroquinolone use and percent resistance in most individual study years (1999–2001 for *P. aeruginosa*, 1999–2002 for *S. aureus*). When the method of generalized estimating equations was used, a population-averaged longitudinal model incorporating total fluoroquinolone use and the previous year's resistance (to account for autocorrelation) did not show a significant effect of fluoroquinolone use on percent resistance for most drug-organism combinations, except for the relationship between levofloxacin use and percent MRSA. The ecologic relationship between fluoroquinolone use and resistance is complex and requires further study.

## Abstract 2

Increasingly resistant bacteria in sickle cell disease patients indicate need to evaluate extendedspectrum cephalosporin therapy.

Antimicrobial drug resistance in bacterial pathogens is of national and international concern (1,2). Although use of antimicrobial agents is accepted as a major driving force behind the spread of resistance, the nature of this relationship is complex (3). Two problematic nosocomial pathogens are *Pseudomonas aeruginosa* and *Staphylococcus aureus*; both often express multidrug resistance. A number of case-control studies at individual hospitals have identified fluoroquinolone use as a risk factor for acquisition of fluoroquinolone-resistant *P. aeruginosa* and methicillin-resistant *S. aureus* (MRSA) (4–8). While outcomes for individual patients are most important from a clinical point of view, an ecologic perspective is also useful to assess the relationship of aggregate antimicrobial drug use to aggregate measures of bacterial resistance. Ecologic investigations across multiple hospitals have reported significant correlations between fluoroquinolone use and percent resistance for MRSA (9) and *P. aeruginosa* (10,11). However, these studies have primarily focused on teaching institutions, used drug expenditure data rather than hospital billing records as a measure of use, or were conducted over a limited time span. We measured fluoroquinolone use as well as the percentages of MRSA and fluoroquinolone-resistant *P. aeruginosa* across 24 US hospitals during a 5-year period. The purpose of this observational study was to determine if volume of aggregate fluoroquinolone use in individual hospitals and bacterial resistance in individual years and during the entire study period are associated.

## Methods

### Participating Hospitals

Hospitals included in this study were participants in the Surveillance and Control of Pathogens of Epidemiologic Importance (SCOPE)–MediMedia Information Technology (MMIT) Antimicrobial Surveillance Network. MMIT (http://www.mminfotech.com) is a healthcare informatics corporation that extracts drug-use data from hospital billing records. Data collection for this project began in 1999 with 19 participating hospitals; that number increased to 48 hospitals in 2003. Of these, 15 hospitals in 1999, 23 hospitals in 2000 and 2001, and 24 hospitals in 2002 and 2003 provided adequate drug-use and microbiology data for inclusion in this study. Demographic data for the hospitals in the year 2002 were obtained from the MMIT database and the American Hospital Directory (http://www.ahd.com). Members of the Council of Teaching Hospitals and Health Systems (http://www.aamc.org/members/coth/start.htm) were designated as teaching hospitals.

## Measurement of Hospital Fluoroquinolone Use

Total grams for each fluoroquinolone used during each year were electronically extracted from individual patient billing records and aggregated to reflect hospitalwide usage. The total number of patient days (PD) for the corresponding time period at each hospital was determined from the sum of individual patient lengths of stay. These data were used to express normalized antimicrobial drug use in defined daily doses per 1,000 patient days (DDD/1,000PD) as recommended by the World Health Organization (WHO) (http://www.whocc.no/atcddd/). The DDDs used were levofloxacin 500 mg, moxifloxacin 400 mg, gatifloxacin 400 mg, and ciprofloxacin 1,000 mg. (Because the MMIT database does not indicate the proportion of intravenous versus oral ciprofloxacin used, we used the oral DDD for ciprofloxacin of 1,000 mg for all ciprofloxacin use).

### Measurement of Hospital Susceptibility for *P. aeruginosa* and *S. aureus*

Hospital antibiograms were requested from each participating hospital for each study year. To be included in the analysis, antibiograms must have reported data on organisms from all clinical sites (i.e., systemic and urinary isolates) and all units (including intensive care units). Ciprofloxacin or levofloxacin susceptibility was used to determine the percentage of fluoroquinolone-resistant *P. aeruginosa*. Oxacillin or nafcillin susceptibility was used to determine the percentage of MRSA.

### Statistical Analysis

Mixed-effects repeated-measures analysis of variance was used to analyze changes in fluoroquinolone use and resistance over the study period, and the Tukey HSD (honestly significantly difference) test was used to compare differences between individual years. Relationships between total and individual fluoroquinolone usage and resistance in target pathogens for each year were determined by univariate linear regression. To analyze the relationship between fluoroquinolone use and percent resistance over the course of the study period, the method of generalized estimating equations (GEE) was used to construct a population-averaged longitudinal model (12). Unlike traditional least squares regression, GEE models do not assume that each observation is statistically independent. Instead, only observations across units are assumed to be independent (i.e., the group of observations from the first hospital are independent from the observations from the second hospital). Other methods, such as generalized least squares, can also account for this correlation of observations within a given unit; however, these methods are based on the assumption that the correlation structure among observations from the same unit is correctly specified. The GEE method provides some protection against this misspecification. This protection can be improved by using a modified sandwich variance estimator to calculate robust standard errors. The model constructed in the current study assumed a first-order autoregressive correlation structure since a 1-year lagged resistance term was used in the model to control for the prior year's resistance. GEE methods also allow data across time points to be analyzed simultaneously, rather than in a year-by-year fashion. Therefore, this GEE model represents the longitudinal change in the outcome in relation to the longitudinal change in the set of predictor variables using all of the available data. A p value <0.05 was considered significant, and all tests were 2-tailed. Because of the exploratory nature of this analysis, p values from univariate linear regressions were not adjusted for multiple testing.

## Results

### Characteristics of Study Hospitals

Table 1 shows the demographic characteristics of the study hospitals in the year 2002. Ten hospitals were designated as teaching hospitals. The location of the hospitals was predominantly from the East (12 hospitals), with 7 hospitals from the South, 3 from the Midwest, and 2 from the West. No significant relationships were seen between resistance in *P. aeruginosa* or percent MRSA in the year 2002 and any of the demographic characteristics by univariate linear regression (p>0.05 for all comparisons).

**Table 1:** Demographic characteristics of study hospitals, year 2002*

Characteristic	Mean ± SD	Median (range)
No. admissions	19,122 ± 12,208	14,720 (5,206–40,676)
No. patient-days	96,488 ± 64,719	76,408 (19,244–219,634)
Case mix index	1.51 ± 0.245	1.52 (1.13–2.01)
Length of hospital stay, d	5 ± 0.67	5 (3.6–6.6)
No. staffed beds	358 ± 203	310 (105–778)
No. intensive care unit beds	22 ± 16	18 (3–80)
No. surgical procedures/1,000 admissions	346 ± 184	281 (163–779)

*SD, standard deviation.

### Fluoroquinolone Use

**Figure 1:**
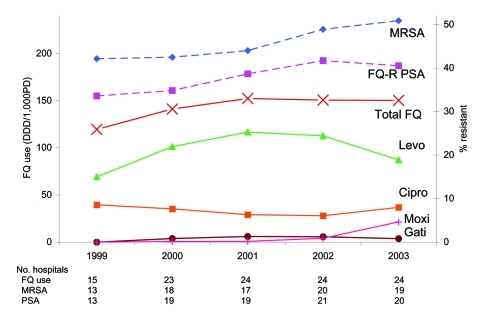
Fluoroquinolone use and resistance over study period. FQ, fluoroquinolone; Levo, levofloxacin; Cipro, ciprofloxacin; Moxi, moxifloxacin; Gati, gatifloxacin; DDD/1,000PD, defined daily doses/1,000 patient-days; FQ-R PSA, fluoroquinolone-resistant *Pseudomonas aeruginosa*; MRSA, methicillin-resistant *Staphylococcus aureus*.

Figure 1 shows changes in the mean of total and individual fluoroquinolone use from 1999 through 2003. For that period, the mean of total fluoroquinolone use increased from 119.6 ± 45.6 DDD/1,000 PD in 1999 to 150.4 ± 44.4 in 2003 (p = 0.011). These changes were driven by the early increase in fluoroquinolone use, as mean values were not significantly different in later (2000–2003) study years (p>0.05, Tukey HSD). Changes in levofloxacin use during the entire study were also significant (p = 0.016), but changes in ciprofloxacin use were not (p = 0.186). Fluoroquinolone use in individual hospitals was significantly correlated with the previous year's use (r>0.75 for all years).

The diversity of use of individual fluoroquinolones in hospitals changed during the study period. Figure 1 shows the mean use of individual fluoroquinolones across all hospitals. The number of hospitals in which a particular fluoroquinolone represented most total fluoroquinolone use also changed. In 1999, levofloxacin use represented >90% of total fluoroquinolone use in 4 (27%) of 15 hospitals; 1 hospital used >90% ciprofloxacin. In 2003, 8 (33%) of 24 hospitals used >90% levofloxacin while in 7 hospitals (30%) moxifloxacin and ciprofloxacin combined to account for >90% of total fluoroquinolone use. The percentage of MRSA and fluoroquinolone-resistant *P. aeruginosa* in 2003 was compared between the 8 hospitals that predominantly used levofloxacin, the 7 that predominantly used moxifloxacin and ciprofloxacin, and the remaining 9 hospitals that used a mixture of fluoroquinolones. No significant differences in mean percent resistance were found between the groups (predominant levofloxacin, predominant moxifloxacin/ciprofloxacin, or neither) for either pathogen in 2003.

### Antimicrobial Drug Resistance

Figure 1 shows the changes in mean percentage resistance for the studied pathogens from 1999 to 2003. Most hospitals reported the number of isolates tested; at least 100 isolates of *S. aureus* and 25 isolates of *P. aeruginosa* were tested per year. From 1999 to 2003, mean percent fluoroquinolone resistance in *P. aeruginosa* increased from 33.6% to 40.5% (p = 0.001). Mean percent MRSA increased from 42.1% in 1999 to 50.9% in 2003 (p<0.0001). Correlations between percent resistance in a given year and percent resistance in the previous year were high for both pathogens (r>0.8).

### Associations between Fluoroquinolone Use and Percent Resistance in Individual Years

The results of the univariate regressions between fluoroquinolone use and resistance are summarized in Table 2. For *P. aeruginosa*, significant relationships occurred between total fluoroquinolone use and resistance in the same year for 1999, 2000, and 2001; the relationship had borderline significance in 2002 (p = 0.0562) and was not significant in 2003. Total fluoroquinolone use was associated with percent MRSA in every year except 2003. Table 2 also shows the results of univariate regression with the individual fluoroquinolones levofloxacin and ciprofloxacin. Levofloxacin use was a significant predictor of fluoroquinolone-resistant *P. aeruginosa* in 1999, 2000, and 2001. Increasing levofloxacin use was significantly associated with increased percent MRSA for all study years except 2003. For ciprofloxacin, a negative slope (increasing ciprofloxacin use associated with decreased percent resistance) was observed for most associations with percent resistant *P. aeruginosa* and MRSA, but none of these associations were significant.

**Table 2:** Associations between fluoroquinolone use and resistance in individual years*

FQ use		FQ-R *P. aeruginosa*			MRSA		
		Total FQ	Levofloxacin	Ciprofloxacin	Total FQ	Levofloxacin	Ciprofloxacin
1999	R^2^	**0.525**	**0.593**	0.233	**0.392**	**0.644**	0.255
	p	**0.008**	**12**	0.116	**0.029**	**0.0017**	0.0937
	n	**12**	**0.003**	12	**12**	**12**	12
2000	R^2^	**0.397**	**0.285**	0.519	**0.617**	**0.617**	0.112
	p	**0.004**	**0.019**	0.348	**0.0001**	**0.0001**	0.175
	n	**19**	**19**	19	**18**	**18**	18
2001	R^2^	0.481	0.335	0.067	**0.318**	**0.32**	0.159
	p	0.001	0.009	0.2821	**0.018**	**0.018**	0.1134
	n	19	19	19	**17**	**17**	17
2002	R^2^	0.178	0.112	0.09	**0.267**	**0.28**	0.145
	p	0.056	0.137	0.184	**0.019**	**0.017**	0.097
	n	21	21	21	**20**	**20**	20
2003	R^2^	0.104	0.012	0.01	0.157	0.02	0.001
	p	0.16	0.641	0.66	0.092	0.538	0.9924
	n	20	20	20	19	19	19

*Linear regression of fluoroquinolone (FQ) use versus percent resistance for hospitals. R^2^, coefficient of determination; n, number of hospitals. **Bold** indicates significant relationships (p<0.05). FQ-R *P. aeruginosa*, fluoroquinolone-resistant *Pseudomonas aeruginosa*; MRSA, methicillin-resistant *Staphylococcus aureus*.

### Modeling of Relationship between Fluoroquinolone Use and Resistance

Table 3 shows the results of population-averaged GEE models using data over the study period from 2000 to 2003. The data from 1999 were not used as an outcome variable because of the lagged resistance term used in the model. After adjusting for the previous year's percent resistance, fluoroquinolone use was generally associated with a small and nonsignificant effect on percent resistance in a given year for either pathogen. Levofloxacin did display a significant contribution to percent resistance in MRSA; the coefficient of 0.012 suggests that an additional 100 DDD/1,000PD would lead to a 1.2% increase in percent MRSA over the previous year (p = 0.033).

**Table 3:** Longitudinal GEE models*

Drug	FQ-R *P. aeruginosa*		MRSA	
	Coefficient	p value	Coefficient	p value
Total FQ				
Previous year's resistance	0.875	<0.001	0.804	<0.001
Total FQ use	0.002	0.883	0.025	0.155
Time	-0.312	0.554	1.04	0.040
Constant	6.75	0.001	4.61	0.058
Levofloxacin				
Previous year's resistance	0.868	<0.001	0.818	<0.001
Levofloxacin use	0.005	0.548	0.012	0.033
Time	-0.317	0.579	1.04	0.041
Constant	6.78	0.001	6.38	0.001
Ciprofloxacin				
Previous year's resistance	0.866	<0.001	0.845	<0.001
Ciprofloxacin use	-0.018	0.226	-0.004	0.848
Time	-0.393	0.475	0.991	0.079
Constant	8.185	<0.001	6.55	0.033

*Association of fluoroquinolone and pathogen resistance over time controlling for prior year resistance. GEE, generalized estimating equations; FQ-R *P. aeruginosa*, fluoroquinolone-resistant *Pseudomonas aeruginosa*; MRSA, methicillin-resistant *Staphylococcus aureus*.

**Figure 2:**
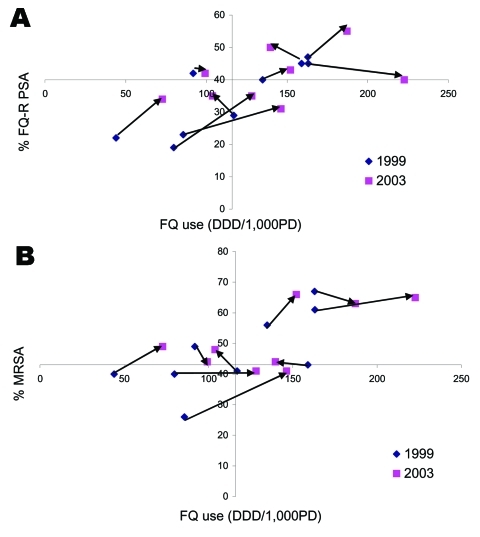
A) Changes in fluoroquinolone use (x axis) and resistance in *Pseudomonas aeruginosa* (y axis) for 9 hospitals with complete data, 1999–2003. Origin is median values of fluoroquinolone use and resistance in 1999. DDD/1,000 PD, defined daily doses/1,000 patient-days. FQ-R PSA, fluoroquinolone-resistant *P. aeruginosa*; MRSA, methicillin-resistant *Staphylococcus aureus*. B) Changes in fluoroquinolone use (x axis) and resistance (y axis) in *S. aureus* for 9 hospitals with complete data, 1999–2003.

Figure 2 shows the baseline-to-endpoint changes in fluoroquinolone use and resistance in *P. aeruginosa* and MRSA for those 9 hospitals with complete data from 1999 to 2003. Fluoroquinolone use increased in 7 hospitals (arrows pointing to the right), and the percentage of fluoroquinolone-resistant *P. aeruginosa* increased in 7 and decreased in 1 (Figure 2A). For the 2 hospitals that reduced total fluoroquinolone use, percent resistance increased slightly in both. The percentage of MRSA increased in 7 hospitals; 5 of these increased fluoroquinolone use and 2 decreased use. The percentage of MRSA decreased in 2 hospitals; both increased quinolone use during the same period (Figure 2B).

## Discussion

The results of this longitudinal, multicenter study of fluoroquinolone use and bacterial resistance suggest a complex relationship between fluoroquinolone use in hospitals and percentage of methicillin-resistant *S. aureus* and fluoroquinolone-resistant *P. aeruginosa* when viewed from an ecologic level. Fluoroquinolone use was linked to greater percent resistance in study years by univariate linear regression. This observation is consistent with the results of other studies linking fluoroquinolone use to resistance in these pathogens (4–11), although the pathways for selection of resistance differ between the 2 pathogens. Fluoroquinolone resistance in *P. aeruginosa* is believed to arise largely from the selection of organisms with point mutations in the topoisomerase enzymes that are targets for the fluoroquinolones (13). This hypothesis is supported by studies demonstrating the emergence of resistance during therapy with fluoroquinolones (4). For methicillin-resistant *S. aureus*, de novo emergence of resistance as seen with *P. aeruginosa* is not a common event (14); rather, patients are generally believed to acquire methicillin-resistant strains of *S. aureus* from the environment (e.g., through cross-transmission); antimicrobial drug use may increase the likelihood of colonization or amplify the resistant population after colonization (15). Fluoroquinolones are not active against most methicillin-resistant isolates (16), providing a selective pressure for MRSA. While any antimicrobial agent that is not active against MRSA should increase a patient's risk for infection, fluoroquinolones may be particularly likely to do so, since they have appear to have unique effects on the expression of MRSA resistance determinants (17) and fibronectin-binding proteins (18).

Using a model incorporating the previous year's percent resistance as well as fluoroquinolone use and time over the entire study period, we did not find an additional effect of total fluoroquinolone use on percent resistance. Many factors beyond the volume of use of an antimicrobial agent affect the emergence and spread of antimicrobial resistance to that drug in the hospital setting. Cross-transmission between patients, acquisition of organisms from the hospital environment, and the use of different antimicrobial agents with linked resistance to the agent under study are all factors that also affect the number of resistant isolates in a given hospital (19–21). These factors are difficult to control for, since quantitative measures of infection control are lacking, and examining the effects of multiple antimicrobial agents complicates analysis. We also did not account for antimicrobial drug use in the community, which we have previously reported to be associated with hospital resistance rates (22). Because of the large number of variables that may influence resistance in the hospital setting, we would expect hospital fluoroquinolone use to have at best a modest effect on resistance. The results of our model suggest that the ecologic effect size of fluoroquinolone use is such that substantial changes in fluoroquinolone use may be required to affect percent resistance. Thus, a larger sample size, which incorporates more hospitals over a greater period, may be required to demonstrate significance, if such effects exist.

Because percent antimicrobial resistance in a given period is highly correlated to previous percent resistance (i.e., the observations are not independent), we incorporated the previous year's percent resistance into our longitudinal model. Failing to account for this autocorrelation may result in spurious associations (23). However, our use of a 1-year lag period was imposed by the nature of our data (which was aggregated on a yearly basis) rather than biologic considerations and may not be the most accurate method of modeling this relationship. We did not time-lag the effect of fluoroquinolone use. Monnet et al. used time-series analysis with autoregressive integrative moving average (ARIMA) functions to model the changes in percent MRSA in a hospital in Scotland during a 3-year period (24). Their transfer function model for predicting percent MRSA used the previous month's percent MRSA; the effect of fluoroquinolone use had a 4-month lag in the effect of changes in fluoroquinolone use on percent MRSA. No study has specifically examined lag effects with fluoroquinolone use and fluoroquinolone-resistant *P. aeruginosa*, although Lopez-Lozano et al. used a lag period of 3 and 5 months in percent resistance and a 1-month lag in ceftazidime use to model the relationship between ceftazidime use and ceftazidime-resistant gram-negative bacilli (25). Thus, use of yearly antibiograms may not be adequate to properly model the relationships between percent resistance in a given year, previous resistance, and fluoroquinolone use. Future studies should attempt to attain more detailed data to allow for more flexibility in modeling the relationship between antimicrobial use and resistance.

The results of this study raise a number of questions for further investigation. Why did the association between fluoroquinolone use and resistance become weaker in the later study years? Does this finding represent random fluctuation or an underlying trend? Mean fluoroquinolone use, after increasing through the first 3 study years, reached a plateau in the last 2 years (Figure 1). Perhaps associations with resistance are reflected most strongly when antimicrobial drug use is increasing, as was seen in the first 3 study years. The study by Zervos et al. (11) found a significant association between changes in fluoroquinolone use and changes in resistance in *P. aeruginosa* in 10 teaching hospitals from 1991 to 2000. During this period, fluoroquinolone use increased by a mean of 97% in the participating hospitals; in the present study, the mean increase during the study period was 23%. Also, the percentage of resistance among MRSA and *P. aeruginosa* was relatively high in all hospitals from the beginning of this study. Monnet et al., in their study of the impact of antimicrobial drug use on an outbreak of MRSA in Scotland, observed that "…antimicrobial drug use was a more important ecologic risk factor at the start of the outbreak than once MRSA had become endemic in the hospital" (24). After antimicrobial use "drives" resistance to a certain point, other effects such as cross-transmission may become the dominant mode of spread. From a scientific standpoint, this finding suggests that ecologic studies may be more likely to detect a significant effect when a particular form of resistance is in its "infancy" (current examples might be linezolid-resistant *S. aureus* or fluoroquinolone-resistant *Streptococcus pneumoniae*). From a clinical point of view, this finding suggests that measures to ensure proper antimicrobial drug use would have the most effect before resistance becomes widespread.

Also of interest is the effect of individual fluoroquinolones on resistance. In our univariate analyses, levofloxacin use had a much stronger association with resistance in both pathogens than did use of ciprofloxacin. Levofloxacin also showed the only significant relationship (with percent MRSA) among the longitudinal models. However, the univariate models did not control for prior resistance levels. Also, the population-averaged modeling approach results in a pooling of effects across all hospitals, resulting in some degree of effect attenuation. Indeed, the estimates from a GEE analysis can sometimes be smaller than those estimates produced from a corresponding mixed-effect model (12). This apparent effect attenuation can be viewed as a trade-off for the reduced vulnerability to model misspecification, as previously discussed. Relationships between individual fluoroquinolone use and resistance should be interpreted with caution, as it is possible that our study is biased (an unknown confounder that is causally associated with MRSA or resistant *P. aeruginosa* occurs more frequently in hospitals that use more levofloxacin). However, other ecologic studies have found similar results. At a single teaching institution in an 8-year period, Mohr et al. observed an association between increasing levofloxacin use and increasing percent resistance in *P. aeruginosa*; this effect was not observed for total fluoroquinolone use or use of other fluoroquinolones (26). An ecologic study across 174 hospitals across 6 years by Bhavnani et al. showed that increasing use (measured as drug expenditures) of levofloxacin and ofloxacin, but not ciprofloxacin, was associated with increased percent resistance to ciprofloxacin in *P. aeruginosa* (10). This ecologic effect might arise from a greater in vitro potential for levofloxacin to select for resistant mutants of *P. aeruginosa*, as Gilbert and colleagues have observed (27). For MRSA, a different relationship between fluoroquinolone use and resistance is relevant, since the emergence of methicillin resistance during therapy is not of concern. When viewed from the perspective of selecting for preexisting methicillin-resistant, fluoroquinolone-resistant *Staphylococcus aureus* from a population that contains both resistant and susceptible isolates, levofloxacin might be more likely to select for the preexisting resistant clones because of its greater activity against the susceptible organisms. In such a scenario, the proportion of resistant isolates (percent resistance) may increase, although the incidence of infections due to resistant bacteria may be relatively stable, as argued by Schwaber et al. (28). Thus, analysis of incidence rates of isolation of resistant organisms may tell a different story from analysis of percentage of resistant organisms. We did not determine incidence rates because some of our study hospitals did not include the number of organisms isolated on their antibiograms. Although percent resistance is most likely of concern to a clinician, the incidence rates determine the overall effect on impact of resistance from an ecologic perspective. Future studies should incorporate resistance rates alongside changes in percent resistance to give the complete picture of the effect of antimicrobial drug use on resistance. The differential effects of individual fluoroquinolones on antimicrobial drug resistance are an important area for future study, as hospitals manipulate their formularies with regard to use of individual fluoroquinolones, often for economic reasons.

This study has a number of limitations. The study hospitals do not represent a random sample of US hospitals; further studies are required to determine whether the results are broadly applicable. We were unable to control for differences between hospitals in their methods of antibiogram construction, including methods and reporting of duplicate isolates, which can affect reported resistance (29), as well as hospital culturing practices (such as MRSA screening). Standardization of methods to report antibiograms has been advocated by the Clinical and Laboratory Standards Institute (formerly NCCLS), but adherence is poor (30). We also were not able to control for differences in infection control measures between hospitals, which are a likely source of variability in the prevalence of resistant organisms. Finally, the associations between antimicrobial drug use and resistance found in ecologic studies such as this may not always coincide with those observed on an individual patient level (31). Case-control studies are more appropriate to quantify the risk associated with antimicrobial drug exposure in individual patients.

Our results suggest that the ecologic relationship between the hospital use of fluoroquinolones and antimicrobial resistance in *P. aeruginosa* and *S. aureus* is complex. Future studies to better define this relationship would be worthwhile, in that they would help hospitals determine where to best invest their resources to reduce the overall impact of resistance in their institutions, whether through enhanced infection control measures or more active antimicrobial stewardship, although both measures are likely important. Meanwhile, judicious use of fluoroquinolones is advocated to prevent the loss of this valuable therapeutic class.
